# Blue Leg: Phlegmasia Cerulea Dolens Secondary to May Thurner Syndrome

**DOI:** 10.7759/cureus.21105

**Published:** 2022-01-11

**Authors:** Usama Talib, Amaar Talib

**Affiliations:** 1 Internal Medicine, University of Kentucky College of Medicine, Lexington, USA; 2 Orthopaedics, Hijaz Hospital, Lahore, PAK

**Keywords:** • may thurner syndrome (mts), phlegmasia cerulea dolens (pcd), mechanical thrombectomy (mt), interventional radiology stent placement, blue leg, blue limb, anticoagulation, thrombosis

## Abstract

Phlegmasia cerulea dolens (PCD) can present as leg pain accompanied by bluish discoloration. It is a limb-threatening emergency that needs to be promptly addressed with anticoagulation with consideration of thrombolytics. We present a case of PCD in an 83-year-old female without obvious risk factor for thrombosis, found to have May Thurner Syndrome (MTS) requiring a chemical and mechanical approach to prevent catastrophic outcomes.

## Introduction

In individuals presenting with leg pain and discoloration, phlegmasia cerulea dolens (PCD), cyanosis with underlying deep vein thrombosis (DVT), must be ruled out as it is a limb and a life-threatening emergency requiring prompt treatment [1]. Being more prevalent in lower extremities and female gender, PCD shares the risk factors for DVT that include pregnancy, recent surgery, and medical conditions predisposing to the formation of thrombus including malignancies and COVID-19 infection [1,2]. Delayed diagnosis can be dangerous and can increase the mortality risk by 25-40% and the risk of requiring amputation by 20 to 50% according to various studies [3]. May Thurner Syndrome (MTS) is an anatomic variant predisposing to clot formation due to compression of the left common iliac vein between the lumbar spine and right common iliac artery [4]. Risk factors for MTS also overlap with those for DVT as well as PCD and include female gender, exposure to radiation, and conditions with increased risk for coagulability. We present a case of PCD in a patient with MTS that was promptly diagnosed and treated. This article was previously presented as a poster in FIT Clinical Decision Making: Vascular Medicine 5 session on March 29, 2020.

## Case presentation

**Figure 1:**
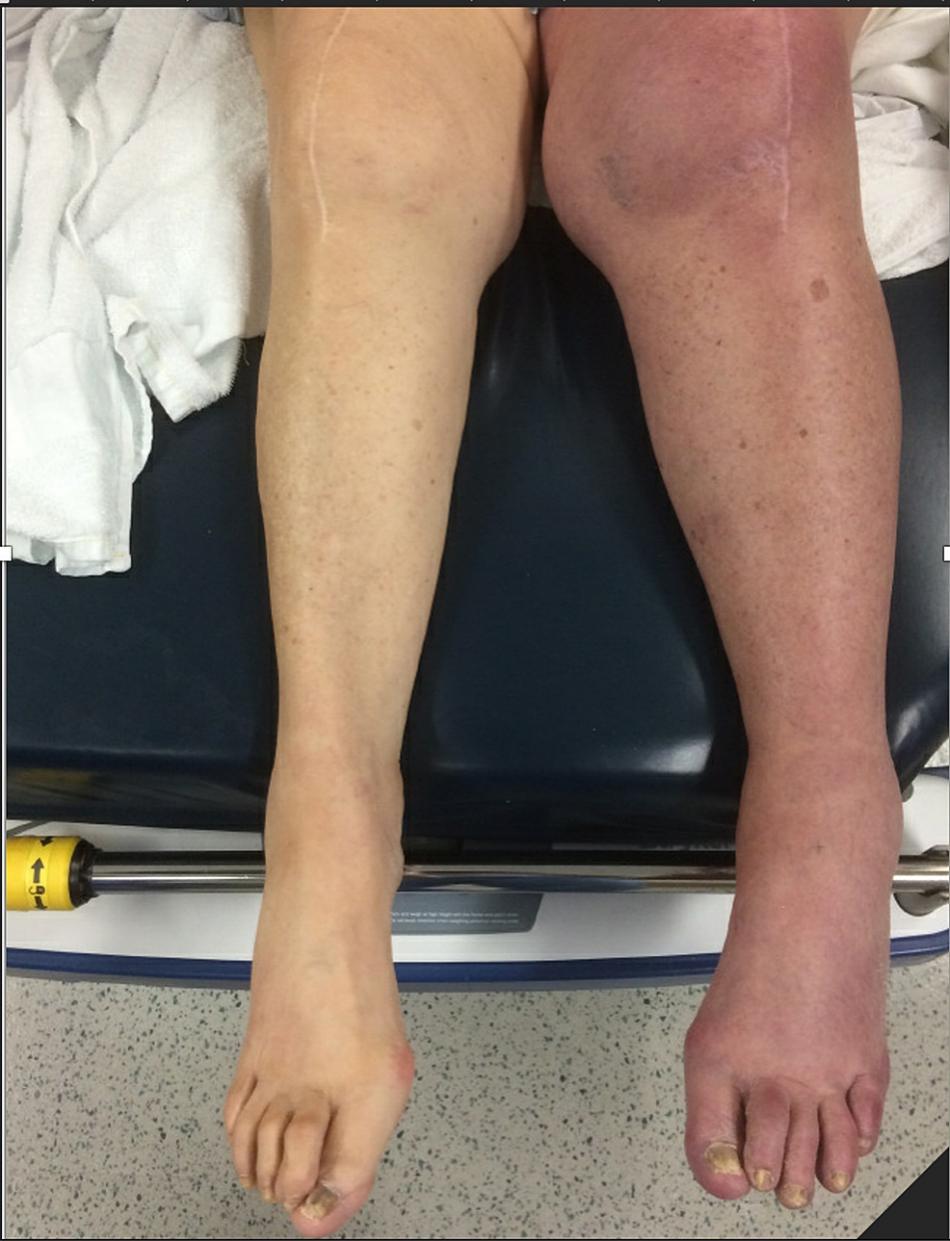
Discoloration of the left leg in the setting of PCD PCD: phlegmasia cerulea dolens

An 83-year-old female with a past medical history of hypertension and type 2 diabetes mellitus presented with one day of worsening left leg pain and swelling. Her pain was dull and constant. The pain started from her groin and had now spread to the whole left lower extremity (LLE) (Figure 1).

**Figure 2:**
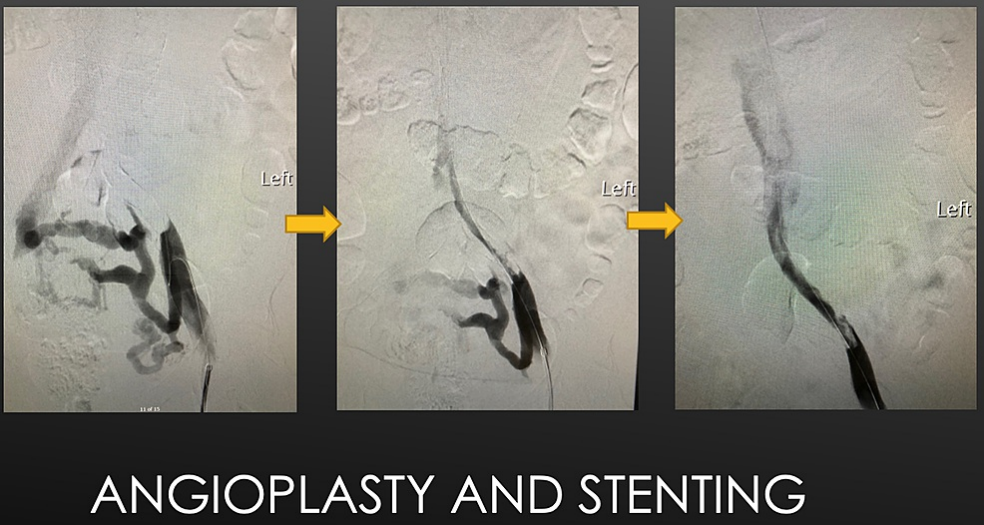
Angioplasty and stenting for PCD in MTS PCD: phlegmasia cerulea dolens; MTS: May Thurner Syndrome

She denied recent trauma, travel, surgery, or hospitalization. She also denied any known malignancy and history of DVT. She denied a history of COVID-19 infection and COVID-19 PCR was negative on admission. Her vital signs were stable. The examination was notable for swollen, cyanosed LLE along with diffuse tenderness with no crepitus on palpation. Sensations in both extremities were intact. 2+ pitting edema was notable on the LLE and pulses were manually palpable bilaterally. Venous duplex ultrasound (VDU) showed extensive LLE DVT with venogram showing severe left common iliac vein stenosis consistent with MTS. She was immediately started on systemic anticoagulation (AC) with un-fractionated heparin. Her management during the hospitalization consisted of inferior vena cava (IVC) filter placement (based on multidisciplinary case review, given extensive DVT with risk for clot migration based on planned interventions) with tissue plasminogen activator (TPA) administration and iliofemoral thrombectomy. Percutaneous angioplasty and stenting of the left iliac vein were done (Figure 2). Her symptoms improved significantly. She was bridged to warfarin prior to discharge with a plan for outpatient follow-up.

## Discussion

MTS predisposes to DVT formation and may manifest as asymptomatic stenosis to severe DVT and limb-threatening ischemia [5]. Management of MTS varies based on the symptoms accompanied at the time of diagnosis. In the absence of contraindications to AC, MTS that accompanies DVT at the time of diagnosis needs a combination of prompt interventions. DVT can lead to various complications if not timely addressed. PCD is a blue discoloration of the extremity with DVT and is associated with significant pain and swelling. Timely identification of PCD is pertinent to initiate systemic AC and is an indication of thrombolysis and/or thrombectomy [6]. It is a part of the spectrum ranging from edema without discoloration (phlegmasia alba), compartment syndrome to gangrene. Cyanosis is pathognomonic for PCD. DVT is usually managed with AC alone [7]. Thrombolysis and/or mechanical thrombectomy is reserved for patients with PCD or extensive iliofemoral DVT as can be seen in DVT associated with MTS [7]. A combination of thrombolysis and endovascular therapy is required as AC alone may not be sufficient to treat PCD in MTS [4,8]. The duration of AC after discharge depends on accompanying risk factors for DVT. Stent placement for MTS has been associated with positive long-term outcomes with 95 to 100% iliac vein patency reported at 2 years [9]. MTS is a rare cause of PCD and requires appropriate time-sensitive management. In the absence of the availability of devices for mechanical thrombectomy, rapid restoration of blood flow may require direct iliac vein stenting, which has been found to be safe and effective in some reports [10].

## Conclusions

Awareness and timely diagnosis of PCD is necessary to ensure prompt intervention to prevent loss of limb or life. A delay in diagnosing and hence treating PCD can increase the risk of requiring amputation by 20 to 50% and the mortality risk by 25 to 40%.

MTS is an anatomic predisposition to DVT formation secondary to external compression of the left common iliac vein leading to stenosis and stasis. MTS is rarely associated with PCD. Management of PCD in MTS requires timely addressing both PCD and MTS with a combination of chemical and endovascular interventions. Duration of AC after discharge is individualized based on coexisting risk factors. Stent placement for MTS has shown encouraging outcomes related to the patency of the iliac vein.
